# Supplementary material for: Genome-wide transcriptomic response of whole blood to radiation
Source: Sci Rep. 2025 Jun 5;15:19840. doi: 10.1038/s41598-025-04898-1 (PMC12141496; doi:10.1038/s41598-025-04898-1)
Supplement: Supplementary file 1 — Supplementary Material 1 [file 41598_2025_4898_MOESM1_ESM.zip › Suppl_rev/Suppl_Fig_S4_rev.pdf]

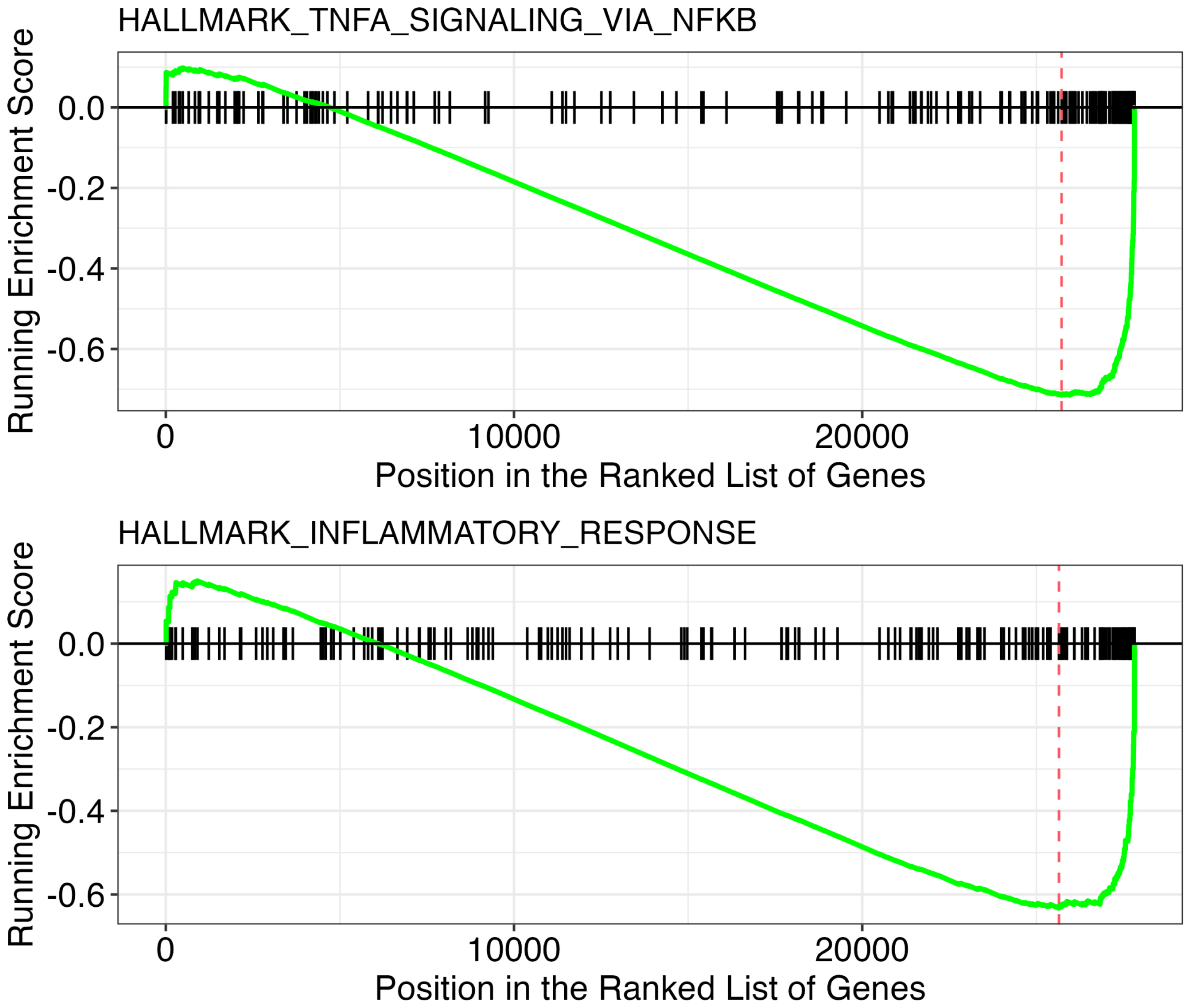

Supplementary Figure S4: Functional annotation by Gene Set Enrichment Analysis with associated normalized enrichment score and p-value. The plot illustrates the transcriptional patters of genes associated with (top panel) TNF signaling via NFKB and (bottom panel) inflammatory response pathways in response to the incubation effect. Genes were ranked by the sign of their LFC multiplied by -log10(pvalue). The corresponding normalized enrichment scores and p-values are provided in Supplementary Table S4.
