# Supplementary material for: Genome-wide transcriptomic response of whole blood to radiation
Source: Sci Rep. 2025 Jun 5;15:19840. doi: 10.1038/s41598-025-04898-1 (PMC12141496; doi:10.1038/s41598-025-04898-1)
Supplement: Supplementary file 1 — Supplementary Material 1 [file 41598_2025_4898_MOESM1_ESM.zip › Suppl_rev/Suppl_Fig_S5_rev.pdf]

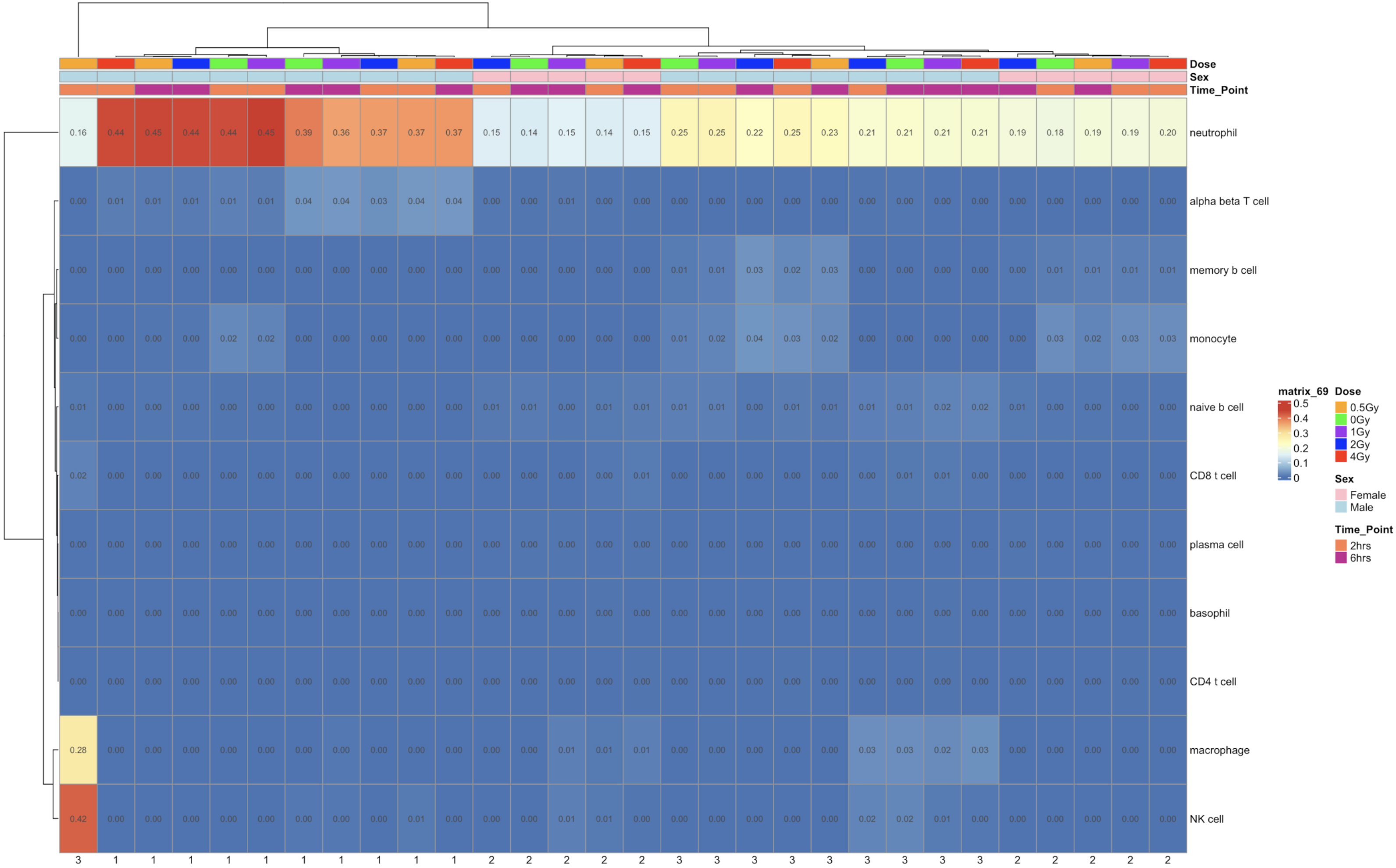

Supplementary Figure S5: Deconvolution heatmap resulting from Omnideconv bayesprism-based estimation of the immune cell type relative abundance.
