# Supplementary material for: Genome-wide transcriptomic response of whole blood to radiation
Source: Sci Rep. 2025 Jun 5;15:19840. doi: 10.1038/s41598-025-04898-1 (PMC12141496; doi:10.1038/s41598-025-04898-1)
Supplement: Supplementary file 1 — Supplementary Material 1 [file 41598_2025_4898_MOESM1_ESM.zip › Suppl_rev/Suppl_Fig_S6_rev.pdf]

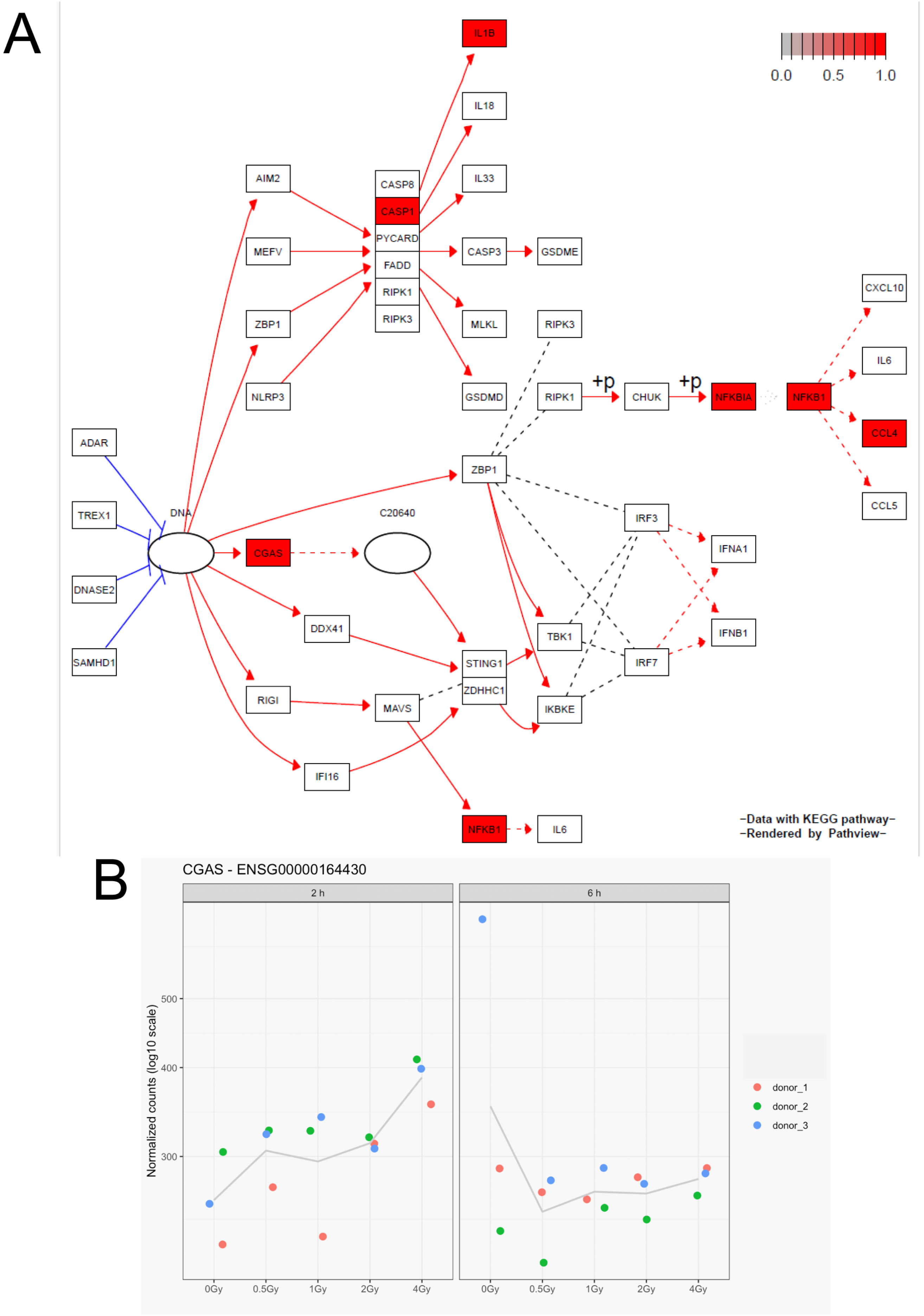

Supplementary Figure S6: (A) cGAS pathway obtained from KEGG (Kyoto Encyclopedia of Genes and Genomes) database and rendered by Pathview. Red-colored genes correspond to differentially expressed genes extracted from 4 Gy vs. 0 Gy comparison 2h post-irradiation. Solid red arrows represent activation of genes, solid blue lines represents inhibition. +P means phosphorylation. (B) Dose-response relationships of normalized cGAS expression 2h and 6h after irradiation.
